# Personalized smart voice-based electronic prescription for remote at-home feedback management in cardiovascular disease rehabilitation: a multi-center randomized controlled trial

**DOI:** 10.3389/fpubh.2023.1113403

**Published:** 2023-06-06

**Authors:** Yin-Hua Zhu, Li-Ping Xia, Jing Yan, Xiao-Ling Shou, Li-Yue Zhu, Yan Sun, Ju-Fei Wang, Xiao-Jun Ji, Mei-Li Zhu, Bei-Li Feng, Hua-Xian Chen

**Affiliations:** ^1^Rehabilitation Center of Zhejiang Hospital, Hangzhou, China; ^2^Department of Cardiology, Shangyu People’s Hospital, Shaoxing, China; ^3^Dean Office of Zhejiang Hospital, Hangzhou, China; ^4^Cardiac Rehabilitation Department of Zhejiang Hospital, Hangzhou, China; ^5^Department of Cardiology, Zhejiang Rongjun Hospital, Jiaxing, China; ^6^Department of Cardiology, Medical Community of People’s Hospital of Fenghua District, Ningbo, China; ^7^Department of Cardiology, Wenzhou Central Hospital, Wenzhou, China; ^8^Rehabilitation Medicine Department of the First People’s Hospital of Yongkang, Jinhua, China; ^9^Department of Cardiology, Ningbo No.2 Hospital (HWaMei Hospital, University of Chinese Academy of Sciences), Ningbo, China; ^10^Department of Rehabilitation Medicine, Xiangyang No.1 People’s Hospital, Xiangyang, China

**Keywords:** cardiorespiratory function, cardiovascular disease, health-related physical fitness, personalized electronic prescription, remote at-home cardiac rehabilitation, risk factors

## Abstract

**Objective:**

To investigate the quality and efficacy of remote at-home rehabilitation for patients with cardiovascular disease (CVD) using personalized smart voice-based electronic prescription, and further explore the standardized health management mode of remote family cardiac rehabilitation. Trial design: A multicenter, randomized (1:1), non-blind, parallel controlled study.

**Methods:**

A total of 171 patients with CVD who were admitted to 18 medical institutions in China from April 2021 to October 2022 were randomly divided into a treatment group (86 cases) and a control group (85 cases) in a non-blinded experiment, based on the sequence of enrollment. The control group received routine at-home rehabilitation training, and the treatment group received remote feedback-based at-home cardiac rehabilitation management based on routine at-home rehabilitation training. The primary outcome was the difference in VO_2_peak (mL/min/kg) after 12 weeks. A linear mixed model was developed with follow-up as the dependent variable. Age and baseline data were utilized as covariates, whereas hospital and patient characteristics were adjusted as random-effect variables. As the linear mixed model can accommodate missing data under the assumption of random missing data, there was no substitute missing value for quantitative data.

**Results:**

A total of 171 participants, with 86 in the experimental group and 85 in the control group, were included in the main analysis. The analysis, which used linear mixing model, revealed significant differences in cardiopulmonary function indexes (VO_2_/kg peak, VO_2_peak, AT, METs, and maximum resistance) at different follow-up time (0, 4, and 12 weeks) in the experimental group (*p* < 0.05). In the control group, there was no significant difference in cardiopulmonary values at different follow-up time (0, 4, and 12 weeks; *p* > 0.05). VO_2_/kg peak (LS mean 1.49, 95%CI 0.09–2.89, *p* = 0.037) and other indicators of cardiopulmonary function (*p* < 0.05) were significantly different between the experimental group and the control group at week 12. The results were comparable in the complete case analysis.

**Conclusion:**

The remote home cardiac rehabilitation management mode using personalized smart voice-based electronic prescription provides several benefits to patients, including improvements in muscle strength, endurance, cardiopulmonary function, and aerobic metabolism. It also helps reduce risk factors for cardiovascular disease and enhances patients’ self-management abilities and treatment compliance.

Clinical trial registration: http://www.chictr.org.cn, identifier ChiCTR2100044063.

## Introduction

Cardiovascular disease (CVD) is a non-communicable disease with a high incidence worldwide (1), affecting 330 million people in China alone (2). Globally, hypertension, the most important risk factor for CVD, affects more than 4.06 billion adults (3). CVD has become a major factor in the global disease burden due to its high morbidity, mortality, and disability rates (4–6). Cardiac rehabilitation (CR) is an integral component in the continuous management of patients with CVD and is often performed in a clinical setting (7, 8). With the ongoing COVID-19 pandemic, at-home CR has become a viable alternative for CVD patients (9). According to the European Guidelines for Prevention of Cardiovascular Diseases (10), at-home CR is expected to see an increase in patient participation, promote positive changes in adverse behavior, and is similar to center-based CR in improving clinical outcomes and life quality (11, 12). There is similar compliance among CVD patients with low to moderate cardiovascular risk (13). Although the remote home CR model is being actively promoted, there are several obstacles, non-compliance with cardiac rehabilitation programs by patients remains a challenge, particularly with respect to the differences in the intensity and timing of exercise, as well as the inability to track progress and effects of rehabilitation programs, creating a further obstacle toward participation in CR. In remote at-home CR, various physiological parameters of patients while they conduct daily activities can be monitored using digital devices. Patients can be directly or indirectly monitored during exercise training and daily activities, ensuring the safe and effective implementation of exercise regimens by patients and improving the overall survival. However, there is a lack of an evidence-based family CR program software platform or smart phone application, as well as relevant assessment tools and standardized rehabilitation management modes, database management support, and ways to ensure the quality and safety of rehabilitation.

This is a multicenter, prospective, randomized controlled study. We referred to the patient assessment recommendations outlined by Balady et al. (14) and the five major prescriptions for cardiac rehabilitation management proposed and applied in the Chinese Cardiovascular Rehabilitation Guidelines (15) (drugs, exercise, smoking cessation, psychology, and nutrition) to develop the study protocol. We evaluated the quality and effectiveness of remote at-home rehabilitation for CVD patients using personalized smart voice-based electronic prescription through the remote at-home feedback management system. Furthermore, we explored the standardized health management mode of remote family cardiac rehabilitation in which both doctors and patients participate.

## Materials and methods

### Trial design

This study was a multi-center, randomized (1:1), non-blinded, parallel controlled study conducted in China (18 medical institutions).

### Research participants

This study is a multi-center clinical study. We enrolled 171 patients who were admitted to 18 medical institutions in China with Zhejiang Hospital as the lead unit, from April 23, 2021 to October 31, 2022. The study protocol was reviewed and approved by the ethics committees of Zhejiang Hospital and its sub-centers, and was registered on the website of China Clinical Trial Registration Center (ChiCTR2100044063, March 9, 2021).

#### Inclusion criteria

(1) ≥ 18-year of age, no gender limitation; (2) Patients were treated for CVD (such as coronary heart disease, post-stenting myocardial infarction, hypertension, peripheral artery occlusion, arrhythmia, chronic cardiac insufficiency, etc.), and the clinical symptoms and cardiovascular-related examination indicators were stable such as, stable angina pectoris, asymptomatic myocardial ischemia, AMI, PCI, or CABG without cardiogenic shock or heart failure, peripheral vascular disease with intermittent claudication, and coronary heart disease risk factors; (3) low risk or intermediate risk in the risk stratification (16); and (4) informed consent.

#### Exclusion criteria

(1) Unstable angina pectoris; (2) patients with systolic blood pressure > 160 mmHg or diastolic blood pressure > 100 mmHg at rest, or blood pressure drops >20 mmHg after standing upright, with symptoms; (3) severe aortic valve stenosis; (4) acute systemic disease or fever; (5) uncontrolled severe atrial or ventricular arrhythmia, uncontrolled obvious sinus tachycardia (>120/min); (6) uncontrolled heart failure, third-degree atrioventricular block without a pacemaker; (7) active pericarditis or myocarditis, thrombophlebitis, recent thromboembolism events, ST segment depression, or elevation (> 2 mm) at rest; (8) severe motor system abnormalities and other metabolic abnormalities, such as acute thyroiditis, hypokalemia, hyperkalemia, or hypovolemia, which can affect exercise capacity; (9) pregnancy; and (10) patients with severe mental illness or dementia.

### Intervention measure

#### The control group received routine at-home rehabilitation training

(1) The patients received health education on cardiac rehabilitation. (2) Cardiopulmonary function, health-related physical fitness, and risk factors were evaluated at the time of enrollment. (3) Individualized exercises for cardiac rehabilitation were prescribed based on the evaluation results: exercise 3–5 times a week, 30 min each time. Exercise methods: jogging or power cycling was used for exercise, low and medium intensity aerobic endurance exercise training, 3–5 times a week, 40 min each training, a total of 12 weeks. The specific methods were as follows: ① 5 min warm-up exercise. Mainly stretching gymnastics and walking. (2) 30 min aerobic training: (a) low intensity aerobic endurance training with target heart rate 40–60% of the maximum heart rate, subjective body sensation calculation RPE < 13 (mild), and 35–45% maximal oxygen uptake; (b) medium-intensity endurance training with the target heart rate 60–75% of the maximum heart rate, subjective body sensation calculation RPE 12–13 (medium), and 46–63% maximal oxygen uptake. ③ 5 min finishing exercise: patients could choose slow walking or relaxation gymnastics. (4) Return to the hospital at 4 and 12 weeks for follow-up evaluation and renewal of exercise prescriptions. The cardiac rehabilitation execution period was 12 weeks, during which 3 evaluations are completed, and at the time of enrollment (week 0), ± 7 days at week 4 and ± 7 days at week 12.

#### Treatment group

Based on the same treatment plan as the control group, the patients were treated using remote feedback at-home cardiac rehabilitation management. (1) The patients were provided with the wearable dynamic ECG recorder [CY-HR-02 (Su Xie Zhi Zhun 20172210861)] of Jiangsu Chuangyue Medical Technology Co., Ltd. for at-home rehabilitation real-time ECG monitoring. The dynamic ECG recorder consists of an ECG signal collector (including a Bluetooth transmission module), a converter, a connecting cable, and the Xinankang™ rehabilitation management app. (2) The patients and their families were instructed on how to use the app to monitor exercises in real time. After the group assessment, the doctors added the patient information on the doctor version of the app and prescribed personalized exercises to the patients, including instructing patients to adjust indicators such as heart rate range, blood pressure, and blood oxygen, and shared exercise training videos, including warm-up, aerobics, resistive exercises, flexibility, relaxation training, etc. (3) The patients used the patient version of the app to connect to the dynamic ECG recorder, and the entire exercise process was managed through smart voice control. The app also monitored the ECG status in real time, provided intelligent warnings for exercise intensity, and helped the patients to maintain their heart rate within the target range. Both doctors and patients could view analysis results in real time, including exercise heart rate, energy consumption (kcal), effective exercise time, and other indicators.

### Indicators and the measurement methods

In this study, indicators were measured three times at 0 (Included baseline value), 4, and 12 weeks, and compare the two groups’ difference between VO_2_/kg peak (mL/min/kg) at week 12 and week 0 was used as the main outcome indicator. The secondary outcome indicators were cardiopulmonary function [including VO_2_peak (mL/min/kg), VO_2_peak (mL/min), AT, (mL/min), METs, and maximum resistance] at 12 weeks, 4 weeks, and 0 weeks. Healthy physical fitness indicators [including grip strength (kg), back grip test (cm), 30-s chair standing test], risk factor indicators [including BMI (Kg/m^2^), neck circumference (cm), and waist-to-hip ratio index] differences; Electrocardiogram (ECG) parameters were used as safety evaluation indicators.

#### Cardiopulmonary exercise testing

Submaximal exercise or symptom-limited exercise was selected as the exercise program, and indicators such as oxygen consumption, 12-lead electrocardiogram, and blood pressure were continuously monitored during exercise. After resting for 3 min, the patients were instructed to cycle at a speed of 55–65 rpm/min for 3 min without load, and then increase the ramp power at a speed of 10–20 week/min until the symptom limit was reached or the target exercise volume reached. The evaluation indicators were VO_2_peak (mL/min), VO_2_/kg peak (mL/min/kg), AT (mL/min), METs, and so on.

#### Risk factor assessment

① Physical assessment: height (m); weight (kg); BMI (kg/m^2^); neck circumference (cm); waist circumference (cm); hip circumference (cm); waist-to-hip ratio. ② Blood indicators: total cholesterol (mmol/L; reference range: 3.0–5.7); triglyceride (mmol/L; reference range: 0.56–1.7); low-density lipoprotein (mmol/L; reference range: 2.1–3.1); high-density lipoprotein (mmol/L; reference range: 1.29–1.55); and fasting blood glucose (mmol/L; reference range: 3.9–6.1).

#### Healthy fitness assessment

① Grip strength test (kg): Muscle strength was assessed using a grip dynamometer; the left and right hands were tested three times each and the best result was taken. ② Back scratch test (cm): The patients were made to stand with their back straight, the right hand placed on the back around the right shoulder with the palm facing the back and the left hand placed on the lower back with the palm facing away from the back. The patients were asked to move the two hands toward each other as much as possible along the spine and to finally touch or overlap each other. When the movement was stable for more than 2 s, the distance between the fingertips of the middle fingers of both hands was measured, and the position of the hand was interchanged for the test. If the fingers of both hands overlapped each other, it was recorded as a positive number, otherwise it was a negative number. This test was used to assess shoulder-dorsal joint flexibility. ③ 30-s chair standing test (times): the number of times the patient could complete the sitting to standing position on a 45 cm-high chair within 30 s. This test assessed muscular fitness.

### Sample size

This study was a randomized controlled trial. The control group received routine at-home rehabilitation training, and the treatment group received remote feedback-based at-home cardiac rehabilitation management based on routine at-home rehabilitation training. The VO_2_/kg peak (mL/min/kg) of the patients was the observed outcome index. According to literature (17–20), the mean value of VO_2_/kg peak in the control group was 19.37 ± 3.20. It is estimated that the VO_2_/kg peak index in the treatment group could be increased by 1.64 compared to the control group after intervention. The sample size was calculated based on the following sample size calculation formula.


$$n=\frac{2{\left(z_{\alpha }+z_{\beta }\right)}^{2}*\sigma^{2}}{\delta^{2}}$$

*n* = 60 cases were obtained. Considering 1:1 randomized grouping and 20% loss of follow-up rate, at least 84 cases in the treatment group and 84 cases in the control group were required, to achieve a total of 168 cases included in the study.

### Randomization

Finally, 171 patients with a definite diagnosis of CVD were included in this study and assigned to the treatment group and the control group using a completely randomized method. Patients meeting the inclusion criteria were numbered sequentially based on the order of enrollment; random numbers were copied from the first row of the random number table and were sorted from lowest to highest. Patients corresponding to even random numbers were placed in the treatment group, and patients corresponding to odd random numbers were placed in the control group. There was no blinding used in the grouping.

### Statistical methods

Data analysis was carried out based on the principle of intention-to-treat (ITT). The Shapiro–Wilk test was used to test the normality of the data. The measurement data conforming to the normal distribution are described by the mean and standard deviation (SD), while those conforming to the skewed distribution are described by the median (interquartile range). Comparing data baselines between groups, the independent sample *t*-test was used for comparison of normal data, and rank sum test was used for skewed data. The categorical data are described using frequencies (number of cases and percentage) and were compared using the *X*^2^ test. SPSS 25.0 software was used for statistical analysis of data. All outcomes of follow-up were used as dependent variables, and a linear mixed model was developed. Baseline measurements and age were used as covariables, and the hospital and study objects were used as random effect factors for adjustment. As the linear mixed model can accommodate missing data under the assumption of random missing data, there was no substitute missing value for quantitative data.

## Results

### Enrollment and loss to follow-up

**Figure 1:**
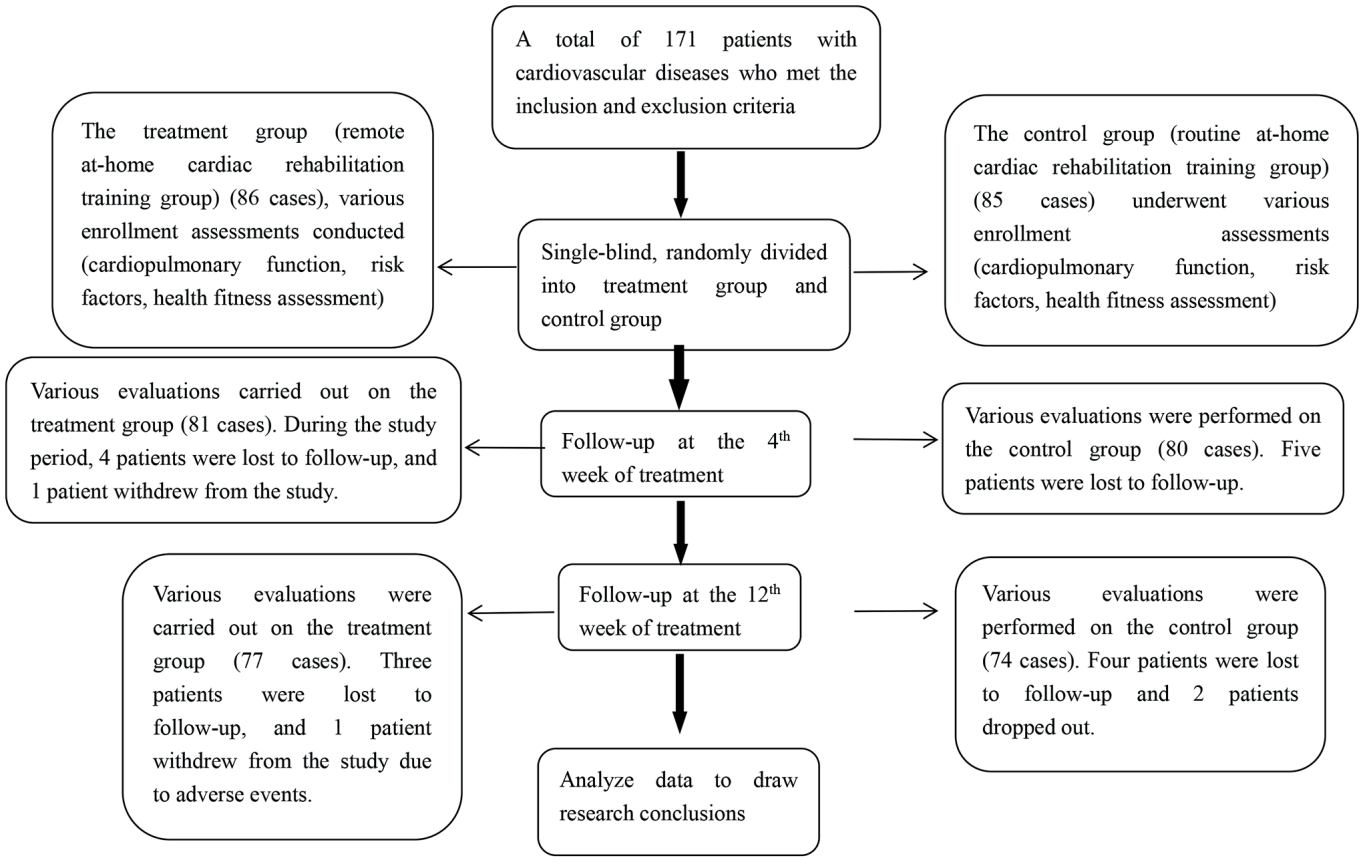
Research flowchart.

In this study, we evaluated 223 patients, including 22 patients who did not meet the inclusion criteria, and 30 patients who met the inclusion criteria but refused to participate in the study. Finally, a total of 171 patients were enrolled and randomly assigned to the control and treatment groups. During the intervention process, patients lost to follow-up were excluded, and patients in the 0-week treatment group (*n* = 86) and the control group (*n* = 85) met the protocol set. Week 4: Treatment group (*n* = 81), control group (*n* = 80); A total of 151 patients completed the 12-week follow-up, including the treatment group (*n* = 77) and control group (*n* = 74). Finally, 86 patients in the treatment group and 85 in the control group were included in the main analysis. Shown in Figure 1.

### Basic information of patients

Before the intervention, there was no statistically significant difference in gender, education level, occupation, smoking, drinking, exercise, obesity, EF value, and blood lipids between the two groups of patients (*p* > 0.05), hence, they are comparable (Table 1). For the bias caused by uneven baseline age, a mixed linear model was used, using age as a covariate to counteract the influence of baseline unevenness to better illustrate the age differences observed in the two groups.

**Table 1:** Comparison of general information of patients.

Comparison of general information of patients			
	NO. (%) or Mean (SD)	Treatment group (*n* = 86)	*p*	Control group (*n* = 85)
*Gender*			0.267
Male	61(71.8)	68(79.1)	
Female	24(28.2)	18(20.9)	
Age	53.81(12.73)	49.68(9.89)	0.019
*Education level*			0.313
University and anove	27(31.8)	37(43.0)	
Middle school	47(55.3)	40(46.5)	
Primary school and below	11(12.9)	9(10.5)	
*Occupation*			0.057
Office work	23(27.1)	33(38.4)	
Heavy physical labor	4(4.7)	0	
Light physical labor	40(47.1)	42(48.8)	
Retired	18(21.2)	11(12.8)	
*Risk factors*			
Smoking/non-smoking	36(42.4)/49(57.6)	38(44.2)/48(55.8)	0.809
Drinking/not drinking	24(28.2)/61(71.8)	22(25.6)/64(74.4)	0.696
*Daily exercise*			0.408
Lack of exercise	32(42.1)	45(52.3)	
Occasional exercise	23(30.2)	20(23.3)	
Regular exercise	21(27.6)	21(24.4)	
*Laboratory data*			
EF(%)	60.91(11.59)	59.73(11.57)	0.545
LDL(mmol/L)	2.68(1.04)	2.74(0.97)	0.686
HDL(mmol/L)	1.08(0.24)	1.13(0.32)	0.245
TC(mmol/L)	4.46(1.37)	4.58(1.27)	0.571
TG(mmol/L)	1.96(1.83)	2.27(2.05)	0.335
Fasting blood glucose(mmol/L)	6.20(2.89)	5.81(1.36)	0.281
SPB(mmhg)	121.58(24.69)	120.96(19.12)	0.888
DBP(mmhg)	75.41(15.67)	78.13(12.45)	0.337
RHR(times\min)	80.53(15.86)	76.49(12.55)	0.159

LDL, low-density lipoprotein; HDL, high-density lipoprotein; TC, total cholesterol; TG, triglycerides; SPB systolic blood pressure; DBP, diastolic blood pressure; RHR, resting heart rate; no statistical difference between the two groups (*p* > 0.05).

### Comparison of cardiopulmonary function indicators

**Figure 2:**
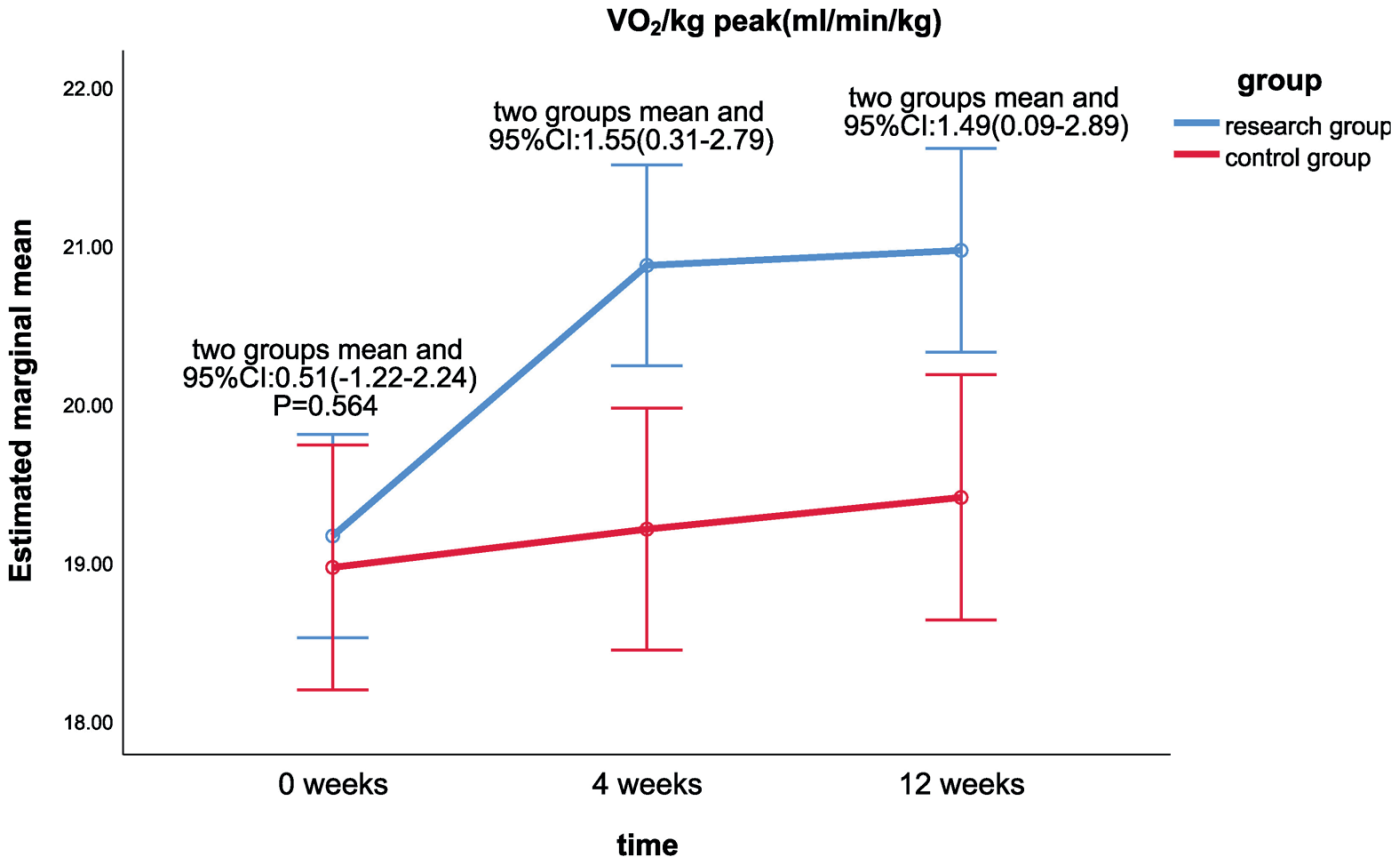
Comparison of VO_2_/kg peak differences between the two groups at week 0, week 4, and week 12.

**Figure 3:**
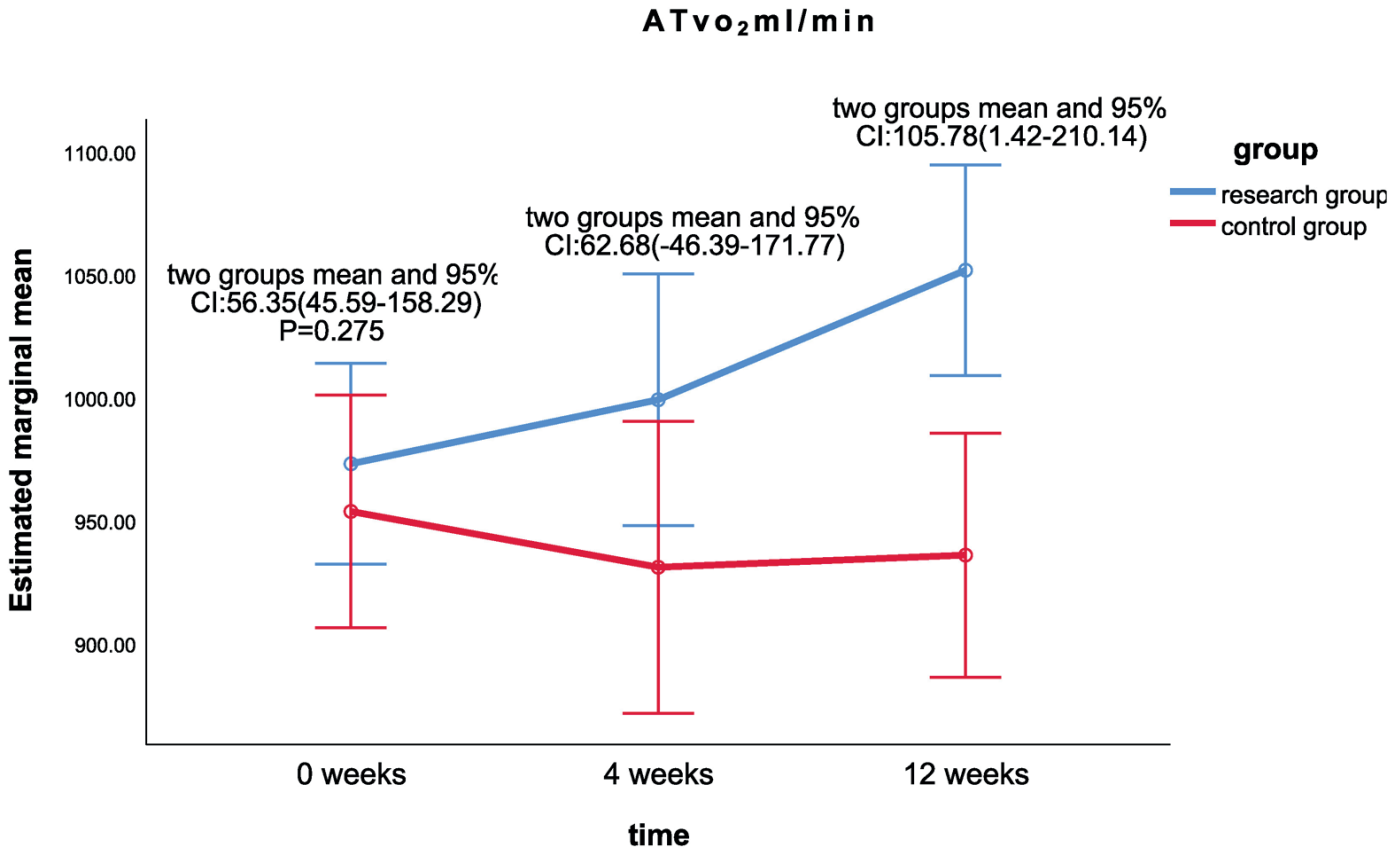
Comparison of ATvo_2_ differences between the two groups at week 0, week 4, and week 12.

**Figure 4:**
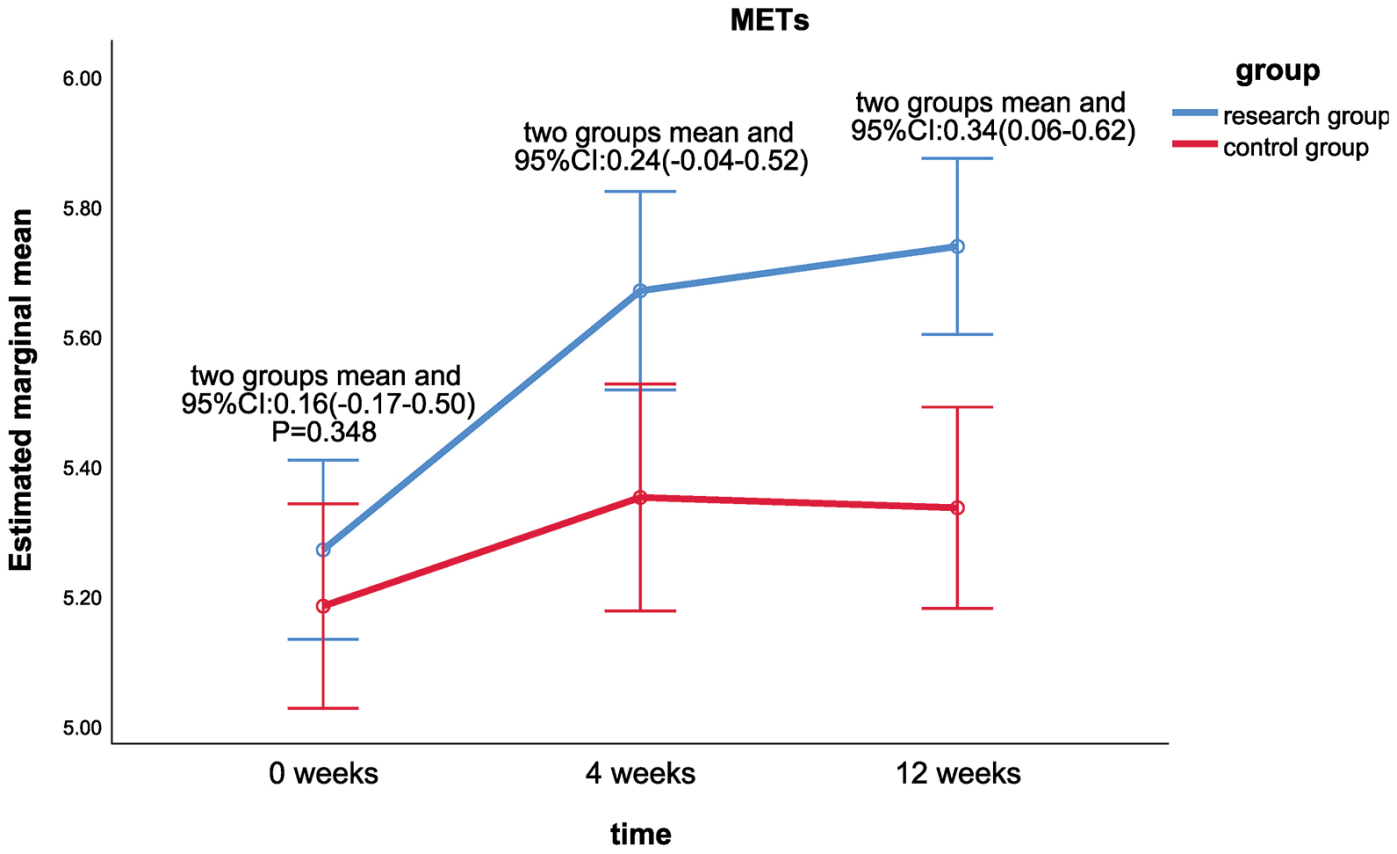
Comparison of METs differences between the two groups at week 0, week 4, and week 12.

**Figure 5:**
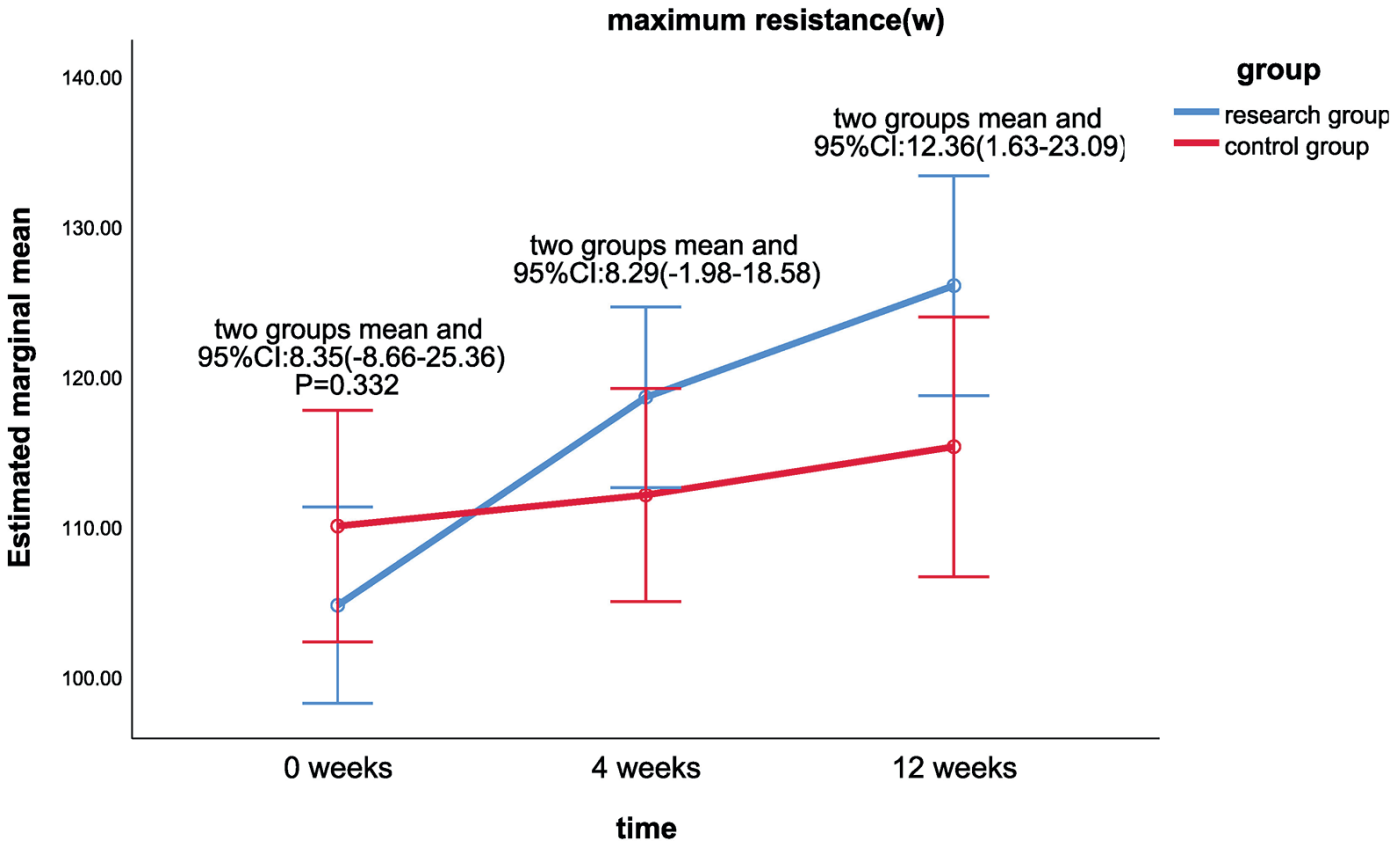
Comparison of maximum resistance (W) differences between the two groups at week 0, week 4, and week 12.

**Figure 6:**
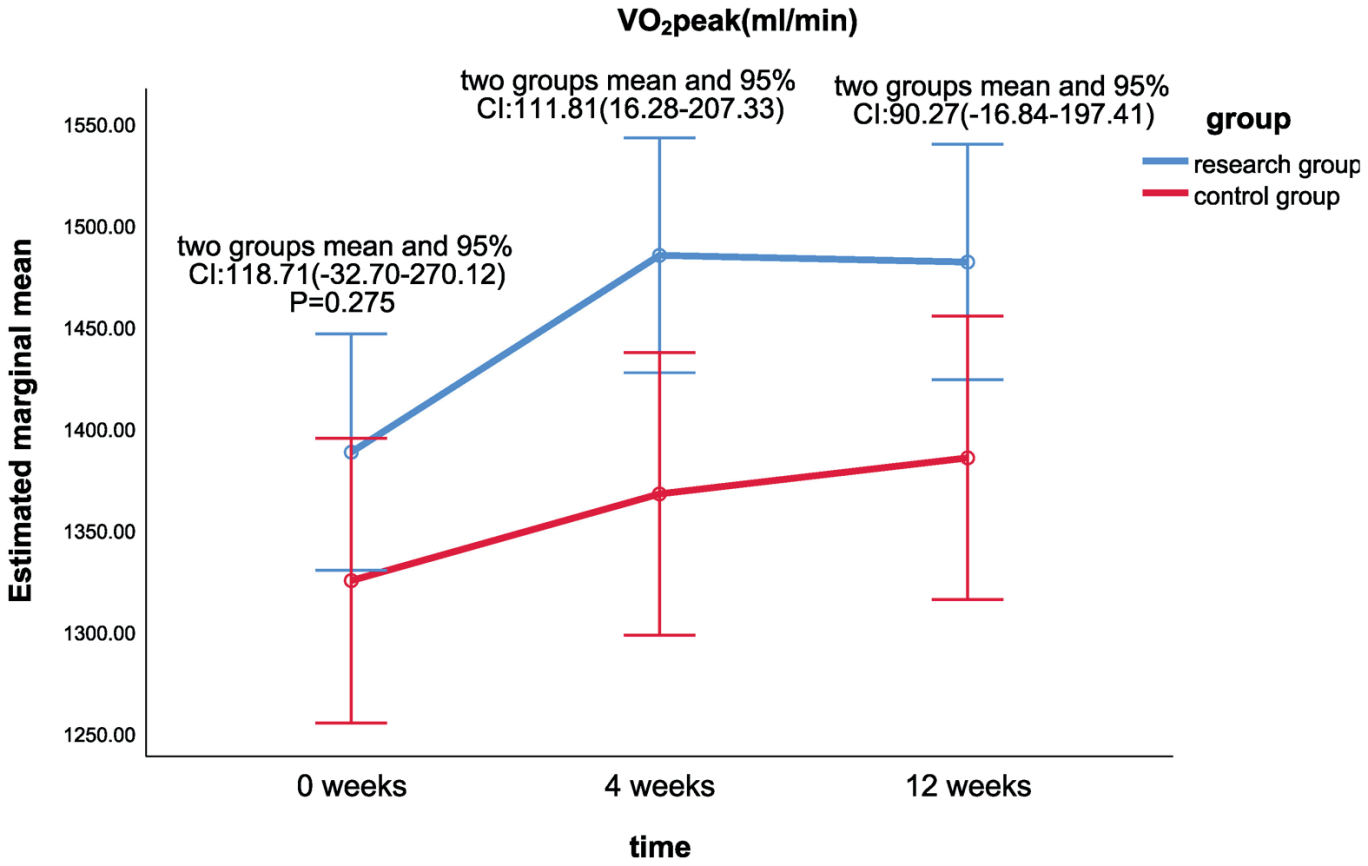
Comparison of VO_2_peak differences between the two groups at week 0, week 4, and week 12.

1. The results of the linear mixed model analysis revealed that the differences of VO_2_/kg peak (*p* = 0.009), VO_2_peak (*p* = 0.025), METs (*p* = 0.018), and maximum resistance-load (*p* = 0.021) at different time points after the intervention were statistically different from the main effect; there was no significant difference in the ATvo_2_ (*p* = 0.063) between the two groups (shown in Table 2). There was no significant difference in the interaction effect of various indicators of cardiopulmonary function (*p* > 0.05; Table 2).
2. Statistically significant differences were found in VO_2_/kg peak (*p* < 0.001), VO_2_peak (*p* < 0.001), ATvo_2_ (*p* = 0.002), METs (*p* < 0.001), and maximum resistance (*p* < 0.001) at different time of 0, 4, and 12 weeks in the treatment group. There was no significant difference in cardiopulmonary values of the control group at 0, 4, and 12 weeks (*p* > 0.05).
3. There was a significant statistical difference between the two groups in the difference between the VO_2_/kg peak (mL/min/kg) values at week 12 and week 0 (*p* < 0.05; LS mean 1.49, 95% CI 0.09–2.89, *p* = 0.037; Figure 2), the respective differences of ATvo_2_ (LS mean 105.78, 95%CI 1.42–210.14, *p* = 0.047; Figure 3), METs (LS mean 0.34, 95% CI 0.06–0.62, *p* = 0.020; Figure 4), and maximum resistance-load (LS mean 12.36, 95% CI 1.63–23.09, *p* = 0.024; Figure 5) at the same two time points have significant statistical differences between the two groups (*p* < 0.05); there was a significant statistical difference between the two groups in the difference between the VO_2_/kg peak(mL/min/kg) values (LS mean 1.55, 95% CI 0.31–2.79, *p* = 0.015; Figure 2) at week 4 and week 0 (*p* < 0.05), so was the difference of VO_2_peak (LS mean 111.81, 95%CI 16.28–207.33, *p* = 0.022; Figure 6) at the same two time points between the two groups.

**Table 2:** Comparison of changes in cardiopulmonary function indicators at 0, 4, and 12 weeks.

VO_2_/kg peak (mL/min/kg)	Baseline	Difference of 4 and 0 week	Difference of 12 and 0 week	*F*	*p*
Research group	19.04(4.37)	1.77(3.56)	1.93(3.55)	11.86	***p*<0.001**
Control group	18.53(4.18)	0.24(2.57)	0.44(3.58)	0.69	0.501
LS mean and	0.51^*^	1.55^#^	1.49^#^		
95%CI	(−1.22–2.24)	(0.31–2.79)	(0.09–2.89)		
*t*	0.57	2.119	2.119		
*p*	0.564^*^	**0.015** ^**#**^	**0.037** ^**#**^		
*VO_2_peak (mL/min)*					
Research group	1397.83(359.86)	106.64(246.89)	112.04(261.05)	8.23	***p*<0.001**
Control group	1279.12(396.55)	14.97(206.99)	55.87(242.15)	1.31	0.275
LS mean and	118.71^*^	111.81^#^	90.27^#^		
95%CI	(−32.70–270.12)	(16.28–207.33)	(−16.84–197.41)		
*t*	1.55	2.328	1.675		
*p*	0.123^*^	**0.022** ^**#**^	0.098^#^		
*ATvo_2_ (mL/min)*					
Research group	979.60(258.35)	45.17(217.28)	87.62(226.33)	4.46	**0.014**
Control group	923.25(230.49)	−5.83(292.40)	−7.58(259.61)	0.04	0.958
LS mean and	56.35^*^	62.68^#^	105.78^#^		
95%CI	(45.59–158.29)	(−46.39–171.77)	(1.42–210.14)		
*t*	1.09	1.143	2.017		
*p*	0.275^*^	0.256^#^	**0.047** ^**#**^		
*METs*					
Research group	5.21(1.04)	0.42(0.86)	0.49(0.87)	18.12	***p*<0.001**
Control group	5.05(1.06)	0.18(0.79)	0.17(0.91)	2.15	0.120
LS mean and	0.16^*^	0.24^#^	0.34^#^		
95%CI	(−0.17–0.50)	(−0.04–0.52)	(0.06–0.62)		
*t*	0.94	1.683	2.364		
*p*	0.348^*^	0.095^#^	**0.020** ^**#**^		
*Maximum resistance (W)*					
Research group	102.60(45.60)	12.53(23.25)	19.51(36.41)	9.99	***p*<0.001**
Control group	110.95(37.75)	2.70(14.77)	5.20(14.79)	3.01	0.056
LS mean and	8.35^*^	8.29^#^	12.36^#^		
95%CI	(−8.66–25.36)	(−1.98–18.58)	(1.63–23.09)		
*t*	0.97	1.595	2.277		
*p*	0.332^*^	0.113^#^	**0.024** ^**#**^		

LS mean, least mean difference. ^#^Calculated based on a linear mixed effects model. ^*^Calculated based on a linear independent samples *t*-test. Statistical results *P* < 0.05 values are shown in bold.

### Comparison of health-related physical fitness

**Figure 7:**
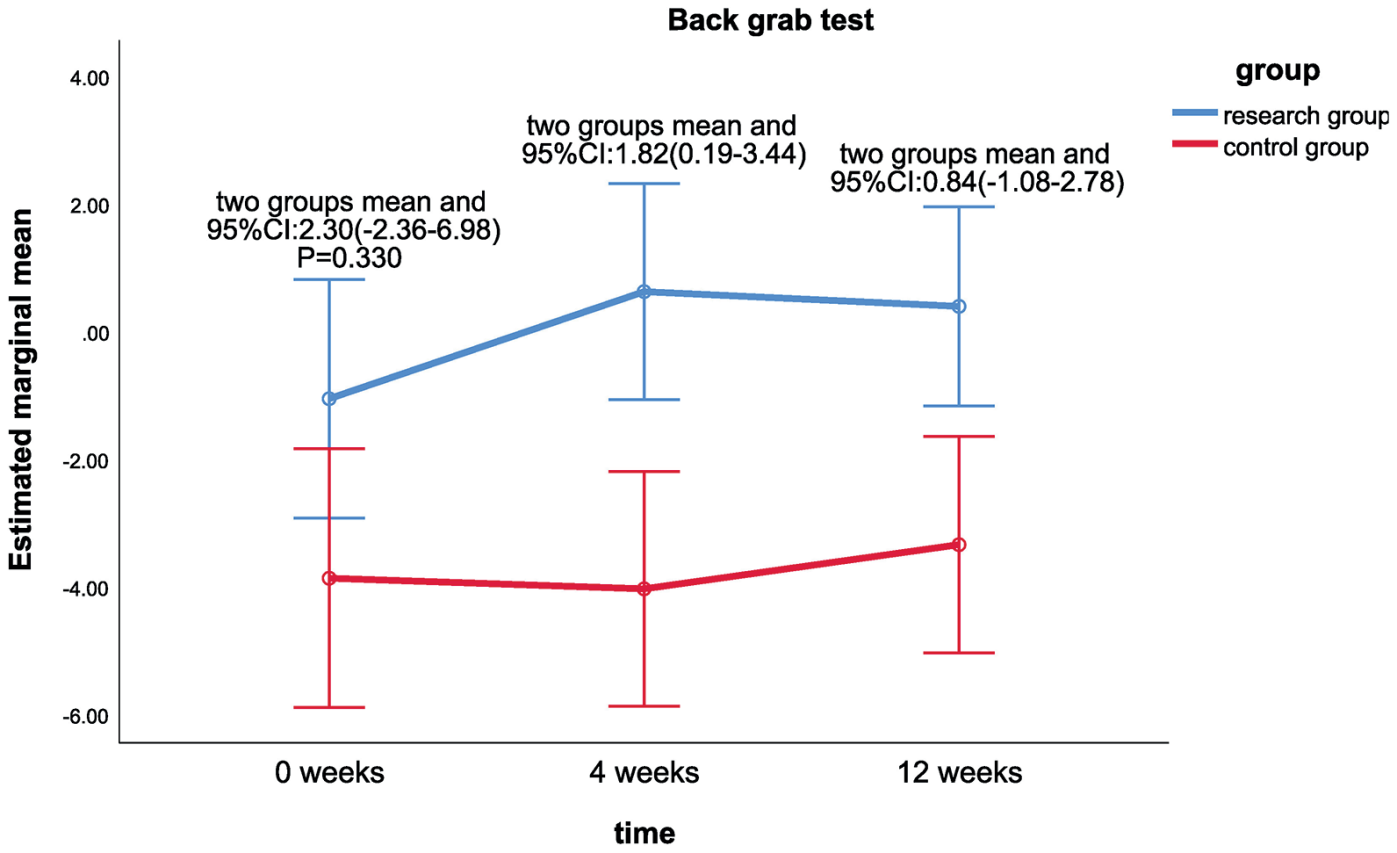
Comparison of back grab test (cm) differences between the two groups at week 0, week 4, and week 12.

1. The linear mixed model analysis showed that there were statistically significant differences between the treatment group at different periods of 0, 4, and 12weeks, grip strength (kg) (*p* = 0.018); (cm; *p* = 0.003) and 30 s (PCS; *p* < 0.001; shown in Table 3). The difference in grip strength of the control group at 0, 4, and 12 weeks (*p* = 0.025) was statistically significant in the 30 s chair standing test (*p* < 0.001).
2. Linear mixed model analysis revealed that there was a statistically significant difference between the groups in the back scratch test between the fourth week and 0 week (LS mean 1.82, 95%CI 0.19–3.44, *p* = 0.028; Figure 7); there was no statistically significant difference between the two groups in the grip strength test between the fourth week and the 0 week (*p* > 0.05), as well as in the 3-s chair stand test between the fourth week and the 0 week.; there was no significant difference between the two groups in the health and fitness indicators between the 12th week and the 0 week (*p* > 0.05). There was no significant difference between the two groups in the interaction effect of various health-related physical fitness indicators (*p* > 0.05; Table 3).

**Table 3:** Comparison of changes in health-related physical fitness indicators at 0, 4, and 12 weeks.

Grip strength test (kg)	Baseline	Difference of 4–0 week	Difference of 12–0 week	*F*	*p*
Research group	34.62(10.03)	1.15(2.46)	1.24(3.52)	4.30	**0.018**
Control group	33.49(10.11)	−0.17(6.55)	2.30(3.61)	3.89	**0.025**
LS mean and	1.12^*^	1.26^#^	−1.24^#^		
95%CI	(−3.19–5.44)	(−1.05–3.58)	(−2.87–0.37)		
*t*	0.52	1.08	−1.53		
*p*	0.604^*^	0.280^#^	0.130^#^		
*Back grab test (cm)*					
Research group	−1.07(11.12)	1.69(3.12)	1.74(5.07)	6.01	**0.003**
Control group	−3.38(13.85)	0.09(5.13)	1.01(6.13)	1.09	0.339
LS mean and	2.30^*^	1.82^#^	0.84^#^		
95%CI	(−2.36–6.98)	(0.19–3.44)	(−1.08–2.78)		
*t*	0.97	2.226	0.869		
*p*	0.330^*^	**0.028** ^**#**^	0.387^#^		
*30-s chair standing test (times)*					
Research group	18.46(6.87)	1.28(3.00)	3.41(4.21)	23.98	***p*<0.001**
Control group	16.37(7.11)	1.66(3.90)	2.95(4.50)	13.78	***p*<0.001**
LS mean and	2.09^*^	−0.28^#^	0.41^#^		
95%CI	(−0.45–4.64)	(−1.65–1.09)	(−1.32–2.14)		
*t*	1.62	−0.406	0.469		
*p*	0.107^*^	0.686^#^	0.640^#^		

LS mean, least mean difference. ^#^Calculated based on a linear mixed effects model. ^*^Calculated based on a linear independent samples *t*-test. Statistical results *P* < 0.05 values are shown in bold.

### Comparison of risk factor indicators

**Figure 8:**
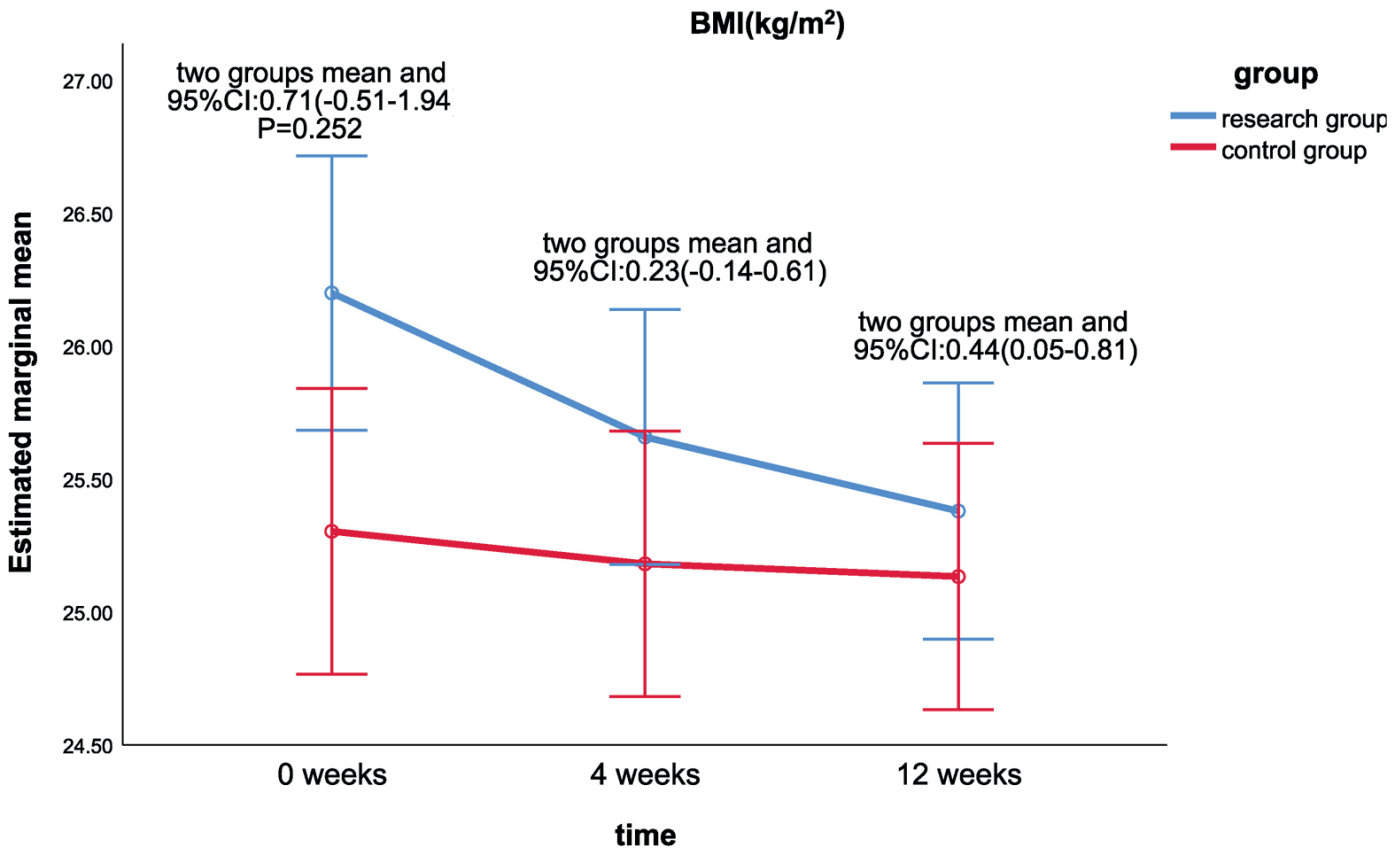
Comparison of BMI differences between the two groups at week 0, week 4, and week 12.

**Figure 9:**
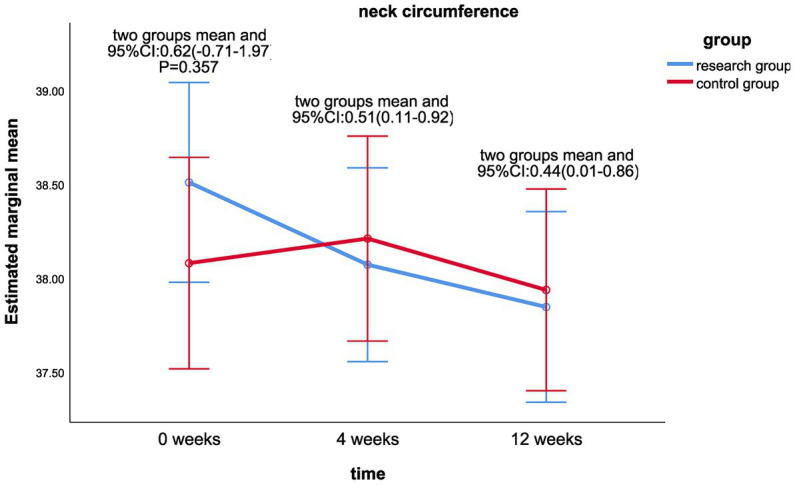
Comparison of neck circumference differences between the two groups at week 0, week 4, and week 12.

1. Linear mixing model analysis revealed that the main effect of cervical circumference (*p* = 0.013) after intervention was statistically different between the treatment group and the control group (shown in Table 4). There were no significant differences in the main effects of BMI, waist-hip ratio, LDL, TC, and TG. There were no significant differences in the interaction effects of risk factors (*p* > 0.05).
2. There were significant differences in BMI (*p* < 0.001), neck circumference (*p* = 0.001), waist-to-hip ratio (*p* = 0.022), LDL (*p* < 0.001), TC (*p* < 0.001), and TG values (*p* = 0.001) at different time of 0, 4, and 12 weeks. LDL (*p* < 0.001) and TC values (*p* < 0.001) were statistically different in the control group at 0, 4, and 12 weeks.
3. There were significant statistical differences in BMI (LS mean 0.44, 95%CI 0.05–0.81, *p* = 0.025; Figure 8) and neck circumference (LS mean 0.44, 95%CI 0.01–0.86, *p* = 0.043; Figure 9) between the treatment group and the control group at week 12 and week 0. There was statistically significant difference between the neck circumference at week 4 and week 0 (LS mean 0.51, 95%CI 0.11–0.92, *p* = 0.014).

**Table 4:** Comparison of changes in risk factor indicators at 0, 4, and 12 weeks.

BMI (kg/m^2^)	Baseline	Difference of 4th–0 week	Difference of 12th–0 week	*F*	*p*
Research group	26.02(3.80)	0.50(0.80)	0.74(1.02)	27.45	***p*<0.001**
Control group	25.31(4.21)	0.13(1.37)	0.23(1.61)	0.97	0.380
LS mean and	0.71^*^	0.23^#^	0.44^#^		
95%CI	(−0.51–1.94)	(−0.14–0.61)	(0.05–0.81)		
*t*	1.15	1.208	2.260		
*p*	0.252^*^	0.229^#^	**0.025** ^**#**^		
*Neck circumference (cm)*					
Research group	38.91(3.41)	0.39(1.13)	0.59(1.22)	7.52	**0.001**
Control group	38.28(3.94)	−0.16(0.92)	0.12(0.89)	2.34	0.102
LS mean and	0.62^*^	0.51^#^	0.44^#^		
95%CI	(−0.71–1.97)	(0.11–0.92)	(0.01–0.86)		
*t*	0.92	2.511	2.049		
*p*	0.357^*^	**0.014** ^**#**^	**0.043** ^**#**^		
*Waist-to-hip ratio*					
Research group	0.93(0.07)	0.003(0.03)	0.016(0.05)	3.95	**0.022**
Control group	0.91(0.07)	0.003(0.02)	0.002(0.02)	0.75	0.474
LS mean and	0.02^*^	−0.004^#^	0.009^#^		
95%CI	(−0.01–0.04)	(−0.02–0.01)	(−0.01–0.02)		
*t*	1.68	−0.744	1.380		
*p*	0.094^*^	0.459^#^	0.171^#^		
*LDL(mmol/L)*					
Research group	2.74(0.97)	0.62(0.77)	0.53(0.91)	20.81	***p*<0.001**
Control group	2.68(1.04)	0.56(0.84)	0.53(1.12)	13.68	***p*<0.001**
LS mean and	0.06^*^	−0.10^#^	−0.05^#^		
95%CI	(−0.25–0.38)	(−0.38–0.18)	(−0.38–0.26)		
*t*	0.405	−0.698	−0.360		
*p*	0.686^*^	0.487^#^	0.719^#^		
*TC(mmol/L)*					
Research group	4.58(1.27)	0.83(0.96)	0.69(1.08)	23.48	***p*<0.001**
Control group	4.46(1.37)	0.69(1.04)	0.59(1.32)	11.62	***p*<0.001**
LS mean and	0.12^*^	0.01^#^	−0.07^#^		
95%CI	(−0.30–0.54)	(−0.33–0.34)	(−0.46–0.31)		
*t*	0.567	0.034	−0.388		
*p*	0.571^*^	0.973^#^	0.699^#^		
*TG(mmol/L)*					
Research group	2.27(2.05)	0.50(1.41)	0.60(1.26)	7.52	**0.001**
Control group	1.96(1.83)	0.38(2.19)	0.32(1.77)	0.88	0.415
LS mean and	0.31^*^	−0.01^#^	0.01^#^		
95%CI	(−0.31–0.92)	(−0.43–0.42)	(−0.32–0.35)		
*t*	0.968	−0.022	0.086		
*p*	0.335^*^	0.983^#^	0.931^#^		

LDL, low-density lipoprotein; TC, total cholesterol; TG, triglycerides; LS mean, least mean difference. ^#^Calculated based on a linear mixed effects model. ^*^Calculated based on a linear independent samples *t*-test. Statistical results *P* < 0.05 values are shown in bold.

## Discussion

The technical equipment currently used in home-based telemonitored cardiac rehabilitation (HTCR) (7) includes human heart rate monitors, training equipment with remote ECG monitoring, smart bracelets, mobile phone applications, real-time telephonic transmission of cardiac rehabilitation monitoring, etc. However, most of the existing studies did not report the details of remote technology, and none of them directly compared HTCR with and without the assistance of technological tools, therefore, it is not possible to fully conclude that the efficacy of the intervention measures is entirely derived from the role of HTCR or is related to some technical factors (21). We adopted the HTCR management mode and quality control for a remote at-home feedback management system using personalized smart voice-based electronic prescription. Through a multicenter, prospective, randomized controlled trial, we investigated and compared two at-home cardiac rehabilitation modes, that is, the at-home cardiac rehabilitation mode under remote monitoring and the conventional at-home cardiac rehabilitation mode without the assistance of technical tools, to provide more evidence for the standardized management mode of HTCR and the efficacy of this mode. Based on the visits at the 4 and 12th weeks, the patients did not suffer any serious adverse cardiovascular event during the at-home cardiac rehabilitation, and the efficacy of the treatment group was better than that of the control group in terms of cardiopulmonary function reserve, risk factor control, and health-related physical fitness. In this study, there were more male cases than female cases, which is consistent with the results of the epidemiological study of cardiovascular diseases by Hu (22).

Peak oxygen consumption, as an evaluation index of cardiopulmonary endurance, can reflect cardiovascular function to a certain extent. Studies have found that for every 1 mL/kg/min increase in peak oxygen consumption during exercise, the risk of cardiovascular disease and all-cause mortality in women and men is reduced by 14–17% (23). The results of our study showed that the cardiopulmonary exercise endurance indexes such as anaerobic threshold oxygen consumption, peak kilogram oxygen consumption, metabolic equivalent, and maximum resistance-load in the treatment group were significantly higher than in the control group at 12 weeks after the intervention (*p* < 0.05). A meta-analysis by Zwisler et al. (24) with a follow-up time of 3 months revealed that HTCR improved peak oxygen consumption (mean difference: 1.6 mL/min/kg). A study by Chen et al. (25) proposed that at-home cardiac rehabilitation increased peak oxygen consumption by 14.2%. In our study, the peak oxygen consumption (mean difference: 1.52 mL/min/kg) increased by an average of 15.4% in this study, similar to their findings. Peng et al. (26) conducted an 8-week at-home cardiac rehabilitation training trial, and the results showed that the quality of life and exercise capacity of the patients in the test group were significantly improved. However, in our study, we found that the peak oxygen consumption of patients increased significantly from the fourth week after the intervention, which is different from previous research results. There are large individual differences in peak oxygen consumption due to different exercise programs and daily activity levels, and different exercise intensity programs cause patients to have different physiological adaptation times. In our study, using real-time monitoring and voice guidance, patients can always maintain the prescribed exercise intensity and ensure effective exercise time. In addition, the rehabilitation management APP can automatically record and feedback the patient’s rehabilitation training data to the doctor, based on which the doctor can adjust the prescribed exercises in a timely manner, to ensure they are more in line with the patient’s real-time tolerance, thus effectively improving the recovery efficiency of the patient while ensuring the safety of exercise training. Aerobic exercise increases skeletal muscle capacity and cardiac output and enhances transport of central O_2_ and utilization of peripheral O_2_, thereby increasing the maximum VO_2_. The results of our study showed that the cardiorespiratory endurance of the patients improved significantly with improvement in skeletal muscle strength at the fourth week, however, the anaerobic threshold oxygen consumption and METs were not improved until the 12th week. This may be related to the fact that the anaerobic threshold can more sensitively reflect the dynamic balance of oxygen supply and demand in muscle tissue during exercise and is less influenced by the subjective effort of patients, power growth rate, and metabolic substrates. With the increase in exercise intensity, the heart rate and blood pressure of the patients gradually increases, which helped cardiomyocytes to adapt to the ischemic environment, increased the ischemic threshold of the heart, increased heart load, and promoted myocardial contractility, and eventually, the length of cardiomyocytes in patients increased (27–29). Oxygen consumption can accurately reflect exercise tolerance (including cardiopulmonary function and skeletal muscle function). The study by Myers et al. (30) showed that the survival rate of cardiovascular disease patients with exercise tolerance <5 METs was significantly lower than that of patients with exercise tolerance >8 METs. Research by Rui (31) found that exercise training can enhance local muscle metabolism, increase the cross-sectional area of type I and type II muscle fibers of skeletal muscle, improve aerobic endurance and physical fitness, and thus improve the daily life quality. Peak oxygen consumption and anaerobic threshold can estimate the maximum cardiac output, assess the degree of cardiac function damage, and determine the cardiac function status of the patients. In our study, based on the Weber classification (32), the cardiac function classes of the two groups were all at class B (VO_2_peak: 16–20, anaerobic threshold: 11–14) at baseline, and the cardiac function classes of the treatment group reached class A from the 4th week after the intervention, however, there was no significant change in the control group.

The back scratch test is a manual assessment method for assessing the flexibility of the shoulder joint and is closely related to the ability of patients to use tools in daily life. Joint flexibility is greatly influenced by body composition such as BMI, abdominal fat area, and muscle level. The results of our study revealed that there was a significant difference in the back scratch test between the two groups in the fourth week after the intervention, but there was no significant difference in the 12th week. As can be seen from Figure 8, the BMI index of the treatment group showed a continuous decreasing trend over time, while no significant change was observed in the control group. In this study, static stretching was the main flexibility training. Some studies have shown (33) that maintaining the pulling state of the skeletal muscle leads to viscoelastic stress relaxation and decreases tensile resistance. However, in human skeletal muscle, this property is short-lived and the improvement in flexibility disappears after 5 weeks. Therefore, flexibility training should focus on the long term. Secondly, this study is a multi-center clinical study, and as the physical fitness was measured in different venues and by different personnel, the possibility of errors cannot be ruled out. Muscle strength is closely related to muscle content and quality, and its reduction reflects the loss of skeletal muscle (34). Studies have found that the reduction of human muscle strength is not significant between the ages of 21 and 40, however, after the age of 40, muscle mass gradually declines with aging, and muscle strength and function decline accordingly (35). Grip strength reflects the strength of the upper body muscles (36). We found that the indicators of muscular fitness (grip strength test and 30-s chair stand test) improved over time, but there was no statistically significant difference between the two groups, which may be related to the older age of the patients in this study (average about 50 years old), and that the at-home rehabilitation training in the study was mainly aerobic exercise focusing on improving cardiorespiratory fitness rather than muscular fitness. Chen et al. (37) did not find simple aerobic exercise to have a significant impact on the grip strength of middle-aged and Older adult people. Relevant studies have shown that resistance training and other exercise interventions can increase muscle strength, muscle endurance, and muscle volume, thereby improving muscular fitness (38–40). We plan to add resistance training in the next study to further observe the rehabilitation effect.

Exercise training can also reduce body weight, improve lipid distribution, reduce total cholesterol levels, and significantly reduce cardiovascular risk factors. The results of our study showed that the BMI index and neck circumference of the treatment group were significantly improved compared with the control group at 12 weeks after the intervention. Although the TG, LDL, and TC values were well controlled and improved over time, there was no significant difference between the two groups, which may be related to the fact that the blood biochemical indicators of the two groups were within the normal range when they enrolled.

To sum up, the standardized remote at-home cardiac rehabilitation management mode using individualized smart voice-based electronic prescription can help patients enhance muscle strength and endurance during exercise, thereby improving the patients’ cardiopulmonary function, aerobic capacity, and body mass index, as well as their self-rehabilitation management ability and treatment compliance, and can form a comprehensive and scientific long-term health management mode in which both doctors and patients actively participate and offer the possibility of tracing the true management of the disease.

Problems encountered in the implementation of tele-home cardiac rehabilitation in this study: (1) Poor patient compliance: ① some patients postponed the visit time or even dropped out; ② poor compliance of patients to exercise prescription: in terms of exercise frequency and intensity, the patients either conducted excessive exercise or reduced the frequency of exercise. For patients in the treatment group, doctors could provide timely reminders and supervision through data feedback, while the control group could not receive objective feedback. ③ During the study, patients wanted to wear exercise monitoring devices to ensure their safety during exercise. However, the wearable devices had issues in terms of comfort and interference from sweat and skin conditions during exercise. (2) The shortcomings of this study are as follows: ① Limited sample size of the research participants: Although this study was a multi-center clinical study, the research time was short, and the sample size was small. In the next study, we will expand the sample size and conduct a systematic and comprehensive intervention. ② The duration of this study was 12 weeks only. Cardiovascular events, death, and hospitalization were not included as endpoints. The duration of the study will be extended to further verify the efficacy of HTCR. ③ The primary types of exercises in this study were low-intensity and medium-intensity aerobic exercises, and the types of exercises were relatively simple. Other types of exercises that are easy to perform should be included in future studies. ④ This study did not investigate satisfaction to different types of family cardiac rehabilitation. A satisfaction survey is planned to be included in future studies to better evaluate the efficacy and effectiveness of different types of cardiac rehabilitation.

## Data availability statement

The original contributions presented in the study are included in the article/supplementary material, further inquiries can be directed to the corresponding authors.

## Ethics statement

The studies involving human participants were reviewed and approved by Ethics Committee of the Zhejiang Hospital. The patients/participants provided their written informed consent to participate in this study.

## Author contributions

Y-HZ, L-PX, JY, X-LS, L-YZ, YS, J-FW, X-JJ, M-LZ, B-LF, and H-XC: conception and design of the research. YS, J-FW, X-JJ, M-LZ, B-LF, and H-XC: acquisition of data. Y-HZ, L-PX, JY, X-LS, and L-YZ: analysis and interpretation of the data and critical revision of the manuscript for intellectual content. Y-HZ and L-PX: statistical analysis. L-YZ: obtaining financing. Y-HZ: writing of the manuscript. All authors contributed to the article and approved the submitted version.

## Funding

This study was supported by the 2022 Basic Public Welfare Research Program Project of Zhejiang Provincial Department of Science and Technology (LGF22H170008).

## Conflict of interest

The authors declare that the research was conducted in the absence of any commercial or financial relationships that could be construed as a potential conflict of interest.

## Publisher’s note

All claims expressed in this article are solely those of the authors and do not necessarily represent those of their affiliated organizations, or those of the publisher, the editors and the reviewers. Any product that may be evaluated in this article, or claim that may be made by its manufacturer, is not guaranteed or endorsed by the publisher.
